# The Diverse Roles of Microglia in the Neurodegenerative Aspects of Central Nervous System (CNS) Autoimmunity

**DOI:** 10.3390/ijms18030504

**Published:** 2017-02-25

**Authors:** Kaitlyn K. Thompson, Stella E. Tsirka

**Affiliations:** Molecular and Cellular Pharmacology Graduate Program, Department of Pharmacological Sciences, Stony Brook University, Stony Brook, NY 11794-8651, USA; kaitlyn.koenig@stonybrook.edu

**Keywords:** autoimmunity, microglia, multiple sclerosis, Rasmussen’s encephalitis

## Abstract

Autoimmune diseases of the central nervous system (CNS) involve inflammatory components and result in neurodegenerative processes. Microglia, the resident macrophages of the CNS, are the first responders after insults to the CNS and comprise a major link between the inflammation and neurodegeneration. Here, we will focus on the roles of microglia in two autoimmune diseases: the prevalent condition of multiple sclerosis (MS) and the much rarer Rasmussen’s encephalitis (RE). Although there is an abundance of evidence that microglia actively contribute to neuronal damage in pathological states such as MS and RE, there is also evidence of important reparative functions. As current research supports a more complex and diverse array of functions and phenotypes that microglia can assume, it is an especially interesting time to examine what is known about both the damaging and restorative roles that microglia can play in the inflammatory CNS setting. We will also discuss the pharmacological approaches to modulating microglia towards a more neuroprotective state.

## 1. Introduction

Microglia, the innate immune cells of the central nervous system (CNS), lie at the intersection of the immune response and the neurodegenerative process—two primary aspects of CNS autoimmune disorders. Autoimmunity is a result of loss of self-tolerance of the immune system, which consequently leads to an immune response against endogenous antigens that are vital to proper organ function. It is generally thought that autoimmune disorders arise in genetically predisposed individuals who encounter an environmental “trigger” [1]. In the context of autoimmune diseases of the CNS, microglia play a central role as the resident “macrophage-like” cells of the brain and spinal cord, rapidly activating upon detection of any pathological insult.

This review will highlight the multiple roles that microglia assume as they contribute to the neurodegenerative process in CNS autoimmune conditions. We will examine this topic in the specific context of multiple sclerosis (MS) as well as discuss possible roles in relation to Rasmussen’s encephalitis (RE). Furthermore, we will explore what is known regarding the neuroprotective functions of microglia and the potential for these cells to be pharmacologically manipulated in order to ameliorate the neurodegenerative process in CNS autoimmune disease states.

## 2. The Numerous Functions and Phenotypes of Microglia

Microglia are the resident innate immune cells of the CNS and are responsible for normal maintenance of CNS tissue as well as the local response to injury or infection, thus playing critical roles in both the healthy and pathological brain and spinal cord. Under homeostatic conditions, microglia vigilantly monitor their microenvironments for detection of injury or infection. This phenotype of microglia, termed “resting” microglia, display a ramified morphology and though they remain in a fixed position within their environment, they are actually quite dynamic cells. “Resting” microglia constantly and rapidly reorganize their processes in order to efficiently scan the microenvironment for insult, whilst their cell body remains stationary so as to not disturb local neuronal circuits [2,3]. Due to this continuous active surveillance, it has been suggested that “resting” microglia may not actually be the most appropriate term [4]. Evidence for other important roles of microglia in the healthy adult brain aside from immune surveillance, such as modulating neuronal activity and removing cell debris and dysfunctional synapses, is also greatly expanding and is reviewed extensively elsewhere [5,6,7].

**Figure 1:**
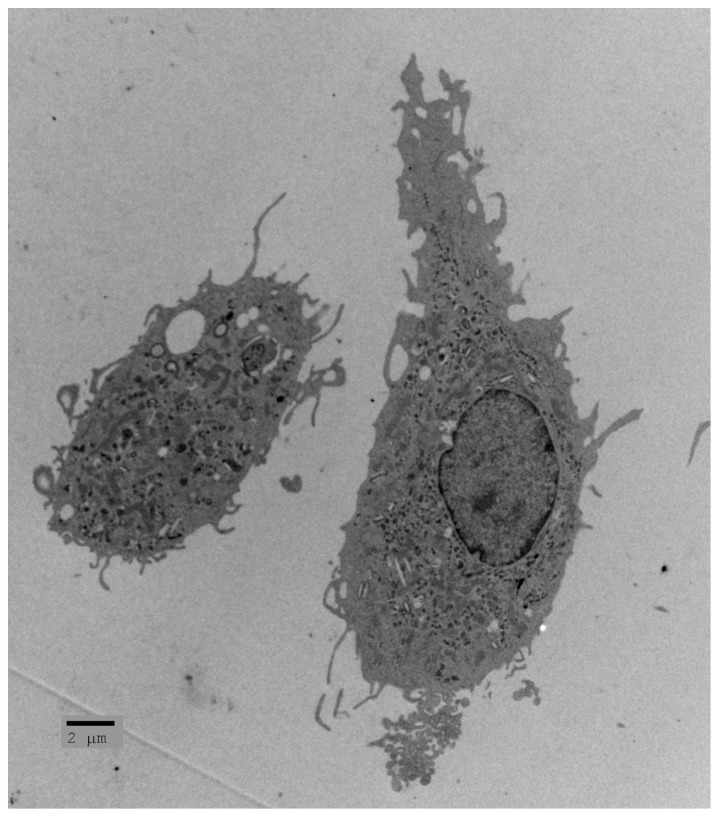
Electron micrograph of microglia. Activated microglia can contribute to neurodegeneration or neuroprotection (courtesy of Dr. Iordanis Gravanis).

**Figure 2:**
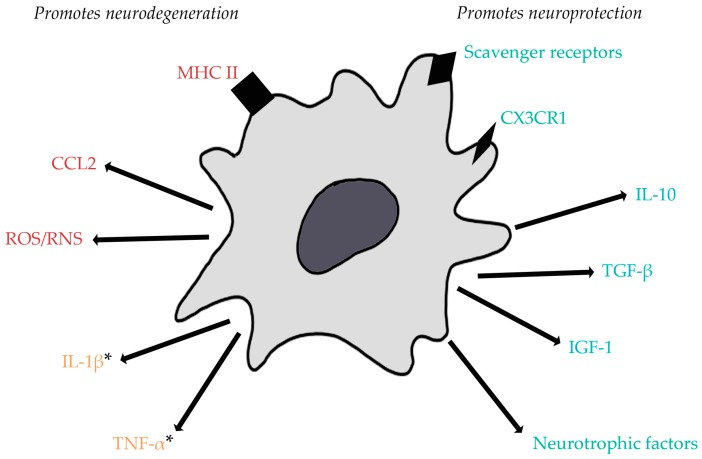
Expression and release of factors by activated microglia can promote neurodegeneration or neuroprotection in situations of central nervous system (CNS) autoimmunity. Generally, pro-inflammatory cytokines and reactive oxygen/nitrogen species contribute to neuronal injury and CC chemokine ligand 2 (CCL2) can recruit immune cells that can be the cause of further neuronal injury. Additionally, upregulation of MHC II promotes efficient antigen presentation and can lead to autoreactive T cell activation. On the other hand, expression of scavenger receptors and the fractalkine receptor (CX3CR1) by microglia promotes phagocytic activity allowing for clearance of dead cells or myelin debris, as seen in multiple sclerosis (MS). Neurotrophic factors, anti-inflammatory cytokines, and insulin growth factor 1 (IGF-1) attenuate pro-inflammatory responses and promote tissue repair. * Though tumor necrosis factor α (TNF-α) has generally been considered more neurotoxic, low levels of this pro-inflammatory cytokine have recently been found to be neuroprotective in cases of excitotoxicity, which occurs in both MS and Rasmussen’s Encephalitis (RE). Both TNF-α and interleukin (IL)-1β have also been seen to be crucial for effective remyelination. ROS/RNS: reactive oxygen/nitrogen species. Red indicates factors that promote neurodegeneration; blue indicates factors that promote neuroprotection; yellow indicates factors shown to play both neurodegenerative and neuroprotective roles.

The roles of microglia as “surveillors” and their responses to pathological situations are their most characterized functions. They are often referred to as the resident macrophages of the CNS. Upon disturbance of tissue homeostasis, the functional phenotype of microglia changes from “resting” to “activated”. Once activated, microglia undergo a dramatic morphological change, withdrawing their processes and assuming an ameboid shape (Figure 1) [3,8]. Along with this change in morphology come drastic changes in gene expression, number, and function. These changes are extremely heterogeneous, as activated microglia can assume numerous phenotypes. These phenotypes have been broadly categorized into M1 (classically activated, pro-inflammatory) and M2 (alternatively activated, anti-inflammatory). The subsets were originally defined in studies of macrophages in vitro [9]. M1 macrophages/microglia were characterized to release pro-inflammatory cytokines, such as interleukin-1β (IL-1β) and tumor necrosis factor α (TNF-α), and reactive oxygen/nitrogen species (ROS/RNS) [10,11]. This phenotype was seen upon activation with interferon-γ or microbial products such as lipopolysaccharide (LPS) [12]. Though the pro-inflammatory mediators released by M1 microglia are beneficial in combatting infection or tumor growth, they can also be the cause of secondary neuronal damage. Therefore, though protective in the cases mentioned above, these pro-inflammatory microglia have generally been considered more harmful to healthy cells. On the other hand, M2 macrophages/microglia were originally described as being activated by IL-4, but were then found to exist on a small spectrum as different stimuli activated the cells to induce slightly different anti-inflammatory phenotypes [12]. These various M2-like cells have been referred to as M2a, M2b, and M2c macrophages/microglia. M2a cells are induced by IL-4 and IL-13 and result in a downregulation of pro-inflammatory mediators along with upregulation of scavenger receptors, factors that provide signals for tissue repair, such as insulin-like growth factor 1 (IGF-1), and arginase 1 (ARG1) which has been considered a typical M2 marker. M2b activation is thought to occur through toll-like receptor 4 (TLR-4) and IL-1R via stimulation with agents such as LPS or IL-1β. These M2 cells produce high levels of IL-10, an anti-inflammatory cytokine, but also release TNF-α, IL-1β, and IL-6 which are all pro-inflammatory. Lastly, M2c cells are stimulated by IL-10 and transforming growth factor β (TGF-β) and downregulate pro-inflammatory cytokines (Figure 2) [12].

Recently, however, it has become clearer that this M1/M2 dichotomy is largely oversimplified [13,14]. A 2016 study utilized single-cell RNA sequencing of individual brain macrophages after traumatic brain injury to clearly illustrate simultaneous expression of classical M1 and M2 markers within individual macrophages in the CNS [15]. These findings, along with others, indicate that these previously described polarized subsets are not so clear-cut. Individual cells may be able to adopt complex phenotypes that exhibit both inflammatory and restorative functions. Additionally, the evidence which points to more distinct phenotypes is mostly from isolated in vitro environments, but the in vivo setting is infinitely more complicated. Examining a wider array of markers and genetic changes in both in vitro and in vivo settings is now more crucial to gaining a better understanding of the numerous microglial phenotypes possible when activated with different stimuli or treated with different pharmacological agents [13,14].

Microglia can also act as antigen presenting cells that communicate with the adaptive immune system within the CNS. Upon activation, microglia upregulate expression of major histocompatibility complex (MHC) molecules and co-stimulatory molecules such as B7 and CD40, allowing them to efficiently present antigens to T cells. Neurotrophins and anti-inflammatory cytokines have been found to inhibit the expression of these surface molecules, indicating that there are regulatory signals to keep the inflammatory, antigen-presenting functions of microglia in check [16]. In the context of CNS autoimmune disorders, on which this review is focused, the expression of MHC and co-stimulatory molecules and the resultant efficient antigen presentation by microglia can promote activation of T cells that recognize CNS antigens and thus, contribute to tissue damage.

Microglia are phagocytic cells and, like macrophages, they are able to eliminate dead cells, bacteria, and other debris. This function has been shown to be extremely important during development and in the healthy adult CNS, as well as more obviously in pathological situations such as infections or neurodegenerative conditions. For instance, in the case of Alzheimer’s disease, microglia can phagocytose toxic amyloid β proteins and in the case of MS, they are able to clear myelin debris and degenerating axons [17,18].

## 3. Microglia Contribute to Neurodegeneration in CNS Autoimmune Disorders

Although microglia “subtypes” are not as distinct as previously described and investigators now must keep in mind the dynamic existence and complex nature of activated microglia, there are undoubtedly phenotypes that can contribute to neurodegeneration or support a more neuroprotective function. These various neurotoxic or regenerative microglia are dependent upon the initial stimulus that results in activation as well as subsequent events.

Microglia-mediated neurotoxicity has been well described in vitro. Stimuli such as LPS, amyloid β, and neuronal injury induce microglia to release pro-inflammatory, neurotoxic factors such as ROS [19]. ROS are generated by nicotinamide adenine dinucleotide phosphate (NADPH) oxidase upon microglial activation with these pro-inflammatory triggers and can be the source of further neuronal damage [20]. Excessive levels of ROS have long been thought to contribute to the pathology of neurodegenerative conditions such as Alzheimer’s disease and Parkinson’s disease [21]. Microglia activated by the aforementioned stimuli also release pro-inflammatory cytokines that further promote inflammatory responses such as IL-1β and TNF-α. TNF-α, though not shown to be directly neurotoxic, has been found to facilitate neurodegeneration via autocrine signaling that stimulates the release of glutamate by microglia, which can promote excitoneurotoxicity [22]. Also, IL-1β has been shown to play a major role in contributing to neuronal loss in excitotoxic conditions [23].

More recently, imaging of microglia in vivo by positron emission tomography (PET) has opened new doors in learning how activated microglia participate in neurodegenerative diseases. Activated microglia can be detected via PET by a selective translocator protein (TSPO) radioligand ^11^C-PK11195. TSPO is normally expressed at low levels in the healthy CNS, but is upregulated by microglia (and astrocytes) upon activation [24]. As the microglia proliferate faster than other CNS cells after injury, the detection of the radioligand indicates the extent of microglial activation [25]. This technique is especially exciting because it can be utilized to track activated microglia throughout disease progression in both rodent models as well as humans. Several imaging studies using ^11^C-PK11195 have been performed in patients with Parkinson’s disease, Huntington’s disease, dementias, and, most relevant to this review, MS. Furthermore, second- and third-generation radioligands, aside from ^11^C-PK11195, are currently being developed and studied in both animal models and in the clinical setting (Table 1). This technique has helped draw the connection between microglial activation and neurodegeneration.

Here, we will review the current knowledge regarding the role microglia play in the neurodegenerative process of the most common CNS autoimmune disorder, MS, as well as the much rarer condition RE.

### 3.1. Multiple Sclerosis

MS is the most common CNS autoimmune disease, affecting approximately 2.5 million people worldwide [30]. It is a chronic, inflammatory, neurodegenerative condition characterized by multifocal lesions in the brain and spinal cord. These lesions consist of inflammation, demyelination, blood–brain barrier disruption, and axonal degeneration and result in clinical symptoms such as pain, spasticity, vision problems, cognitive issues, and paralysis. The disease affects females more frequently than males, and diagnosis usually occurs between the ages of 20 and 40, making it one of the leading causes of non-traumatic disability among young adults [24,30,31].

In approximately 85% of cases, MS presents as a relapsing-remitting disease course (RRMS) consisting of abrupt symptomatic episodes followed by periods of variable recovery. However, over time, majority of these patients develop progressive neurological disability during a secondary progressive phase (SPMS) [31,32,33,34,35]. On the other hand, a small percentage (~15%) of patients are diagnosed with primary progressive MS (PPMS) where relapse and recovery phases are rare or nonexistent, and neurological disability steadily accumulates from the time of onset [31,33,35]. The permanent neurological damage evident in PPMS and SPMS is thought to reflect neurodegenerative processes. However, the underlying mechanisms of degeneration in this disease remain poorly understood.

Some studies claim that axonal degeneration is a result of inflammation [36] and/or chronic demyelination [37,38]. Furthermore, it has been suggested that the degenerative process is amplified by brain ageing and accumulating disease burden over time [39]. However, on the other hand, MS has more recently been regarded as a primarily degenerative condition that then results in neuroinflammation [35,40]. Despite this controversy of whether neurodegeneration occurs from the “outside-in” with inflammatory destruction of axons, or the “inside-out” with primary cytodegeneration evoking an autoimmune response, it is undeniable that microglia play a role during this process.

Whether RRMS, SPMS, or PPMS, microglial activation is a hallmark of the disease. Though the very first cellular events of MS still remain elusive, it is reasonable to hypothesize that microglial activation does play a role in the initial pathological process, as these cells are sensitive to even minor insults. It has been suggested that microglia nodules observed in normal-appearing white matter (NAWM) could be one of the earliest events in MS lesion development [41,42]. The microglia present in these “preactive” lesions have been found to express NADPH oxidase, indicative that the microglia in these clusters are likely involved in the production of harmful ROS that, as previously mentioned, can contribute to neuronal damage [42]. Importantly, one study draws the connection between evident neuronal damage and microglial activation and found that the clusters of microglia are associated with damaged axons as indicated by intra-axonal amyloid precursor protein (APP) accumulation and changes in neurofilament phosphorylation [43]. Other work examining a large sample of multiple sclerosis autopsies found a highly significant association between general inflammation as well as the presence of microglia/macrophages and axonal injury, especially in the progressive stage of the disease. Supporting their conclusion that inflammation and neurodegeneration are linked, they also found that in the very late stages of the disease, inflammation declines and axonal injury becomes similar to age-matched controls, indicating that one does not seem to proceed without the other [44].

Imaging studies in patients with MS, using the TSPO radioligand ^11^C-PK11195, have also shown that microglial activation occurs early in the disease process and it has been linked to disability and brain atrophy [27]. A recent study using a second-generation TSPO PET tracer, ^11^C-PBR28, found that radioligand uptake was greater across the brain in SPMS patients as opposed to those with RRMS. Furthermore, increased microglial activation, as indicated by ^11^C-PBR28 binding, in the cortex, deep gray matter, and NAWM correlated with increased neurological disability (Table 1) [29].

Microglia (and macrophages) have also been revealed to be damaging cell types by the most popular animal model for MS: experimental autoimmune encephalomyelitis (EAE). This immune-mediated model of demyelination mimics several aspects of the human disease after rodents are immunized with myelin peptides. Several studies have shown that inhibiting microglial activation delays onset or attenuates the severity of EAE. Popovic et al. illustrated that minocycline, a tetracycline with antimicrobial properties, dramatically suppressed disease activity by inhibiting microglial activation in a chronic EAE model in which rats are injected with myelin oligodendrocyte glycoprotein (MOG) [45]. Similar results were obtained by Heppner et al., who generated an inducible model of microglial inhibition by treating transgenic CD11b-HSVTK mice with ganciclovir. Here, they also saw that ‘paralyzing’ microglia and inhibiting their activity suppressed MOG-EAE symptoms as well as delayed disease onset. Aside from the reduction in CNS inflammation, this attenuation was accompanied by significantly less axonal damage [46]. Further supporting the neurotoxic capabilities of microglia, in a relapsing-remitting EAE model that utilizes proteolipid-protein (PLP) immunization, chronic microglial activation in the cortices was associated with cortical callosal neuronal dysfunction characterized by the loss of expression of synaptic proteins and alterations in axonal transport [47]. TSPO-PET has also been performed in rodents with EAE. One study found that radioligand binding reached maximal values in both the white and gray matter during the peak of clinical disease, but only returned to normal in the white matter, not the gray matter, after recovery [25].

There is also a lot of support for the idea that the stimulus that activates microglia plays a role in their activated phenotype, and in the way they affect the degeneration process. It has been proposed that microglia that are activated by factors released from degenerating neurons secrete neurotoxic molecules which promote further neurodegeneration, forming a positive feedback loop (as described earlier in the case of neuronal injury inducing ROS production by microglia) [13,48]. Therefore, if in fact the “inside-out” hypothesis is true, initial axonal degeneration could promote a microglial phenotype that then goes on to damage the neurons from the outside in as well.

The recruitment of blood-derived monocytes/macrophages into the CNS is also a hallmark of MS and, along with microglia, monocytes/macrophages have been considered a detrimental cell type in this autoimmune setting, promoting demyelination and axonal damage. Normally, monocytes are restricted from entering the CNS, but in pathological conditions they are able to cross the blood–brain barrier and migrate to areas of injury [49]. Animal models of demyelination have illustrated heavy recruitment of peripheral monocytes to lesion areas that is dependent upon chemokine receptor type 2 (CCR2). In a toxin-induced model of demyelination, it was shown that peripheral monocytes lacking CCR2 were unable to infiltrate the CNS, but this did not impact the demyelination or remyelination process [50]. On the other hand, in a model of autoimmune-mediated demyelination (EAE), it was seen that monocyte infiltration correlated with disease progression to the point of paralysis and when *Ccr2*^+/+^ mice were transplanted with CCR2-deficient peripheral blood and bone marrow, they failed to develop the same degree of functional deficits [51].

Microglia express the ligand for CCR2, monocyte chemoattractant peptide-1 (MCP-1/CCL2), and expression of this chemokine has been found to be increased in animal models of demyelination as well as in MS lesions [52,53]. One study observed that CCL2 production by microglia and astrocytes is responsible for directing leukocytes to sites of axonal injury. When entorhinodentate axotomy was performed in CCR2-deficient mice, neither macrophages nor T cells were recruited to the injury site [54]. CCL2 can also function to generally activate microglia, with no bias towards pro- or anti-inflammatory dominance [55]. This has detrimental implications in MS/EAE as recruitment of leukocytes and macrophages as well as further microglial activation can induce neuronal damage. In fact, CCL2 levels have correlated with severity of relapses in PLP-EAE [56] and CCL2-blocking antibodies protect against EAE [57].

### 3.2. Rasmussen’s Encephalitis 

Rasmussen’s encephalitis (RE) is a rare, progressive, CNS autoimmune disorder that usually occurs in childhood or young adulthood. However, unlike MS where genetic susceptibility is a likely factor in its development, RE is considered a sporadic disease with no genetic basis and an etiology that remains elusive. Clinically, RE manifests as intractable epilepsy and progressive deterioration of neurologic functions in one affected brain hemisphere [58]. This condition is characterized by severe cortical inflammation and neuronal loss in the affected hemisphere, thus we will discuss evidence of a role for microglia in the inflammatory and neurodegenerative processes of RE.

RE pathogenesis was initially linked to autoantibodies against the ionotropic glutamate receptor subunit 3 (GluR3) [59]. This finding drove further investigations into antibody-mediated autoimmune mechanisms in RE. However, there has been conflicting evidence, as not all RE patients were found to be positive for anti-GluR3 antibodies in their sera [60] and non-RE epileptic patients also sometimes harbored these antibodies [61,62]. Moreover, when rabbits were immunized with GluR3, some developed inflammation in both brain hemispheres as opposed to the uni-hemispheric inflammation seen in RE patients, and some did not develop epileptic seizures at all which is a prominent characteristic of this condition in humans [59].

More recently, cytotoxic CD8+ T lymphocytes gained attention as a major pathogenic cell type in RE as large numbers of T cells have been observed in the brains of RE patients. A study by Bien et al. performed immunohistochemical evaluation of 11 RE brain specimens and found that CD8+ T cells are at least partially responsible for neuronal cell death. Here, they not only observed that activated CD8+ T cells were present close to MHC class I-positive neurons, but the T cells also contained granzyme B, indicating their mechanism of cytotoxic attack [63]. A more recent 2016 study examined 23 brain, blood, and cerebrospinal fluid (CSF) samples from RE patients and found that peripheral CD8+ T cell expansion correlated with disease severity [64]. This finding supports the suggestion that, in RE, CD8+ T lymphocytes expand in the periphery and mount an antigen-specific attack within the CNS. However, what triggers this expansion is still unknown. 

Examining the role of microglia in this degenerative autoimmune disorder is interesting because their precise pathogenic role is not completely understood, yet reactive microgliosis is a well-founded hallmark of RE. Banati et al. utilized TSPO-PET imaging and found widespread microglial activation in the affected hemisphere of RE patients [26]. A more recent study utilized ionized calcium binding adaptor molecule 1 (Iba1), a calcium-binding protein upregulated upon activation, and immunohistochemistry, and found extensive microglial activation in RE specimens as well as an array of microglial morphologies, indicating complex and diverse reactivity [65]. However, in this same study, there was no significant correlation between CD8+ T cell infiltration and microglial reactivity, leading the investigators to speculate that independent mechanisms are triggering these different inflammatory processes [65]. 

In terms of how these, possibly distinct, inflammatory processes contribute to neurodegeneration, a comprehensive study that examined the pathology of 45 hemispherectomies drew a connection finding that axonal injury in the white matter was associated with the presence of inflammatory cell infiltrates [66]. This study by Pardo et al. also suggested a possible progression of events within RE as early morphological findings included T lymphocyte infiltration in the CNS as well as activation of astrocytes and microglia. However, at these early time points of the disease there is little evidence of neuronal damage, indicating that the inflammatory cells, including microglia, are likely responsible for causing the injury [66]. 

More is known about the role microglia play in situations that involve excitotoxicity, such as epilepsy. It could be speculated that microglia assume similar roles in RE as in epilepsy, as epileptic seizures are a characteristic of this condition. For instance, Vezzani et al. used kainate to induce electroencephalographic seizures in rats and found that this excitotoxic insult stimulated microglia to produce IL-1β, a pro-inflammatory cytokine found to enhance the induced seizures and contribute to neuronal loss [23]. The role for IL-1β was supported in a later study by Ravizza et al. which showed that inhibition of caspase-1 reduced the release of IL-1β, resulting in a delay in seizure onset and reduction in seizure duration [67]. In fact, there is recent support for a pathogenic role of this cytokine, released from microglia, in RE as well. Ramaswamy et al. showed that IL-1β immunoreactivity was found in brain tissue from RE patients and colocalized with MHC II, indicating it was present in microglial cells [68]. A different study found that levels of TNF-α, another pro-inflammatory cytokine released from microglia, in the CSF were elevated at all stages of RE progression [69]. 

Currently, the most effective treatment for RE is hemispherectomy [58]. However, there are several agents believed to be possible candidates for effective RE treatment, including inhibitors of microglia such as minocycline and drugs targeting excitotoxicity like cyclooxygenase-2 (COX-2) inhibitors [58]. Recently, a TNF-α blocking antibody was evaluated in RE patients and found to decrease seizure frequency as well as neurocognitive decline [70], indicating that hindering pro-inflammatory responses including those of microglia might be a valid approach. However, further investigation into causes and pathogenesis of RE are necessary to guide research into possible future treatment strategies. 

## 4. The Neuroprotective Potential of Microglia 

Neuroinflammation is becoming more commonly referred to as a “double-edged sword” in several scenarios. Microglia, a central participant in CNS inflammation, are described above as a major contributor to the neurodegenerative process. However, there are circumstances in which the inflammatory functions of microglia are beneficial for recovery and can promote neuroprotection. Additionally, microglia can assume neuroprotective phenotypes where they release anti-inflammatory mediators and growth factors. 

There are cases when pro-inflammatory factors released from microglia aid in neuronal survival. A recent study examined the role of microglia during excitotoxicity, as commonly seen in epilepsy, Rasmussen’s encephalitis, and other neurodegenerative conditions, in hippocampal slice cultures. Here, it was shown that release of the pro-inflammatory cytokine TNF-α from microglia was required to protect neurons from excitotoxicity, as both the absence of microglia and the presence of a TNF-α-neutralizing antibody significantly increased neuronal death [71]. Furthermore, they found that ATP mediated this release by binding to the P2X purinoceptor 7 (P2X7 receptor), a purinergic receptor present on microglia [71]. TNF-α is also thought to play a reparative role in demyelinating conditions such as MS. A seminal study by Arnett et al. showed that mice lacking TNF-α underwent delayed remyelination compared to wild-type mice after demyelination induced by oral exposure to cuprizone, a copper-chelating agent toxic to oligodendrocytes. This impairment in the remyelinating process was correlated with decreases in oligodendrocyte precursor cell (OPC) proliferation and decreased numbers of mature oligodendrocytes capable of myelination [72]. Similar results were obtained in a study with IL-1β-deficient mice. Here, it was reported that when *IL-1β*^-/-^ mice were fed cuprizone, remyelination upon cuprizone withdrawal was dramatically reduced compared to wild-type mice [73]. This decrease in repair was shown to be due to failure of OPC differentiation into myelinating oligodendrocytes. Additionally, this study observed a lack of IGF-1 within demyelinated lesions of IL-1β-deficient mice, showing a link between IL-1β, the production of IGF-1 by microglia/macrophages, and the differentiation of OPCs into myelination-capable oligodendrocytes [73].

On the other hand, as discussed previously, microglia are able to release anti-inflammatory cytokines, such as TGF-β and IL-10, that are able to suppress pro-inflammatory responses. Both IL-10 and TGF-β have inhibitory effects on harmful RNS that can cause damage to neurons [74]. In an EAE study it was noted that IL-10-deficient mice developed more severe disease compared to wild-type mice illustrating the importance of this anti-inflammatory cytokine in limiting CNS inflammation [75]. Additionally, IL-10 can provide neurotrophic support to injured neurons. For instance, it has been seen to induce NGF production by astrocytes [74,76]. TGF-β also mediates neuroprotective mechanisms in EAE and in excitotoxic conditions, as is seen in both MS and RE. Boche et al. demonstrated that TGF-β1 promotes neuronal survival in kainate-induced excitotoxicity and documented increases in TGF-β1 when cerebral tolerance was induced via LPS injection prior to kainic acid injection. This protective effect of the LPS pre-treatment was ablated when a TGF-β1-blocking antibody was also administered [77]. The evidence that highlights neuroprotective functions of pro-inflammatory and anti-inflammatory factors released from microglia further supports the complexity of microglial activation and the numerous phenotypes they can assume under stimulation with different signals.

Microglia can also produce neurotrophic factors such as brain-derived neurotrophic factor (BDNF), glial cell line-derived neurotrophic factor (GDNF), and nerve growth factor (NGF) [78]. These are considered neuroprotective as they support functional maintenance of neurons and have been shown to play a role in neuronal recovery post-insult. Neurotrophins, such as NGF, have been found to inhibit the expression of MHC II on microglia directly via the p75 neurotrophin receptor [79]. This decreases microglia’s potential for antigen presentation, which, in the context of a disease like MS, can limit their role in facilitating the inflammatory cascade. Furthermore, anti-inflammatory cytokines, such as IL-10 have been seen to induce NGF synthesis [76].

The phagocytic function of microglia is also considered a neuroprotective mechanism, especially in demyelinating, neurodegenerative conditions like MS. The presence of myelin debris inhibits remyelination [80] and thus, debris removal is critical in order to facilitate the remyelination process and, ultimately, protect axons. Importantly, a recent study by Lampron et al. showed that phagocytosis of myelin debris by microglia is impaired in mice lacking the fractalkine receptor (CX3CR1), a chemokine receptor expressed by microglia, in the cuprizone model of demyelination. The authors used electron microscopy to visualize the absence of endosomes, indicating lack of myelin phagocytosis, in CX3CR1^−/−^ microglia. The failure to remove myelin debris subsequently resulted in inefficient remyelination in the corpus callosum after withdrawal of cuprizone [50]. Furthermore, this study linked the impaired phagocytic ability of microglia in CX3CR1^−/−^ mice to triggering receptor expressed on myeloid cells-2 (TREM2) signaling.

TREM2 is expressed on the surface of microglia and plays an important role in their migration and phagocytic activity [81]. Takahashi et al. showed that knocking down TREM2 in microglia impairs clearance of apoptotic neurons and increases gene expression of pro-inflammatory mediators that can contribute to neuronal damage [81]. Examining the role of TREM2 in EAE, Piccio, et al. demonstrated increased expression in the spinal cord due to microglia/macrophage infiltration during both the induction phase and the chronic phase of EAE. Additionally, this study noted that administration of a TREM2-blocking antibody prior to EAE induction resulted in a more severe and chronic disease course [82]. Another study by Takahashi et al. transplanted myeloid precursors overexpressing TREM2 into EAE animals at the peak of the disease and this resulted in improved myelin debris removal as well as increased expression of anti-inflammatory cytokines and decreased expression of pro-inflammatory cytokines in the spinal cord [83]. Thus, there is mounting evidence that TREM2 stimulation leads to induction of a more neuroprotective, anti-inflammatory environment permissive of remyelination.

### Pharmacological Approaches to Inducing a Neuroprotective Microglial Response

Modulating microglia with pharmacological agents to promote neuroprotection is an area that is being heavily focused on in current investigations. As mentioned above, if microglia assume a more anti-inflammatory phenotype, this could induce a more neuroprotective environment in an inflammatory CNS setting, such as in MS. For example, the tetrapeptide, tuftsin (TKPR), has been seen to attenuate EAE by acting specifically on microglia and promoting an anti-inflammatory phenotype [84,85]. We have shown that tuftsin signals, through its receptor neuropilin-1, present on microglia to induce canonical TGF-β signaling, resulting in the release of the anti-inflammatory cytokines TGF-β and IL-10, both of which have been seen to promote neuroprotection [86]. 

Unfortunately, tuftsin is not currently an US Food and Drug Administration (FDA)-approved agent, but several MS drugs that are currently on the market have also been seen to modulate microglial activity to inhibit neurotoxic activity. Glatiramer acetate, approved over 20 years ago for the treatment of RRMS, decreases the secretion of TNF-α and increases secretion of IL-10 as well as phagocytosis by microglia in vitro [87]. It was also found in a TSPO-PET study that one-year treatment of RRMS patients with glatiramer acetate resulted in reduced radioligand binding in the cortical gray matter and cerebral white matter, indicating a possible reduction in neuroinflammation [28]. Fingolimod phosphate (FTY720), a sphingosine 1-phosphate receptor (S1PR) agonist approved for the treatment or RRMS in 2010, was also found to inhibit the production of the pro-inflammatory cytokines TNF-α, IL-1β, and IL-6 by primary microglia and increase the production of neurotrophic factors such as BDNF and GDNF [88]. 

In the previously mentioned study by Masuch et al. supporting the role for microglial TNF-α release as a neuroprotective mechanism during excitotoxicity, they also took a pharmacological approach to promote this function using valproic acid. Valproic acid is an FDA-approved drug used to treat epileptic seizures, but has recently gained attention as a possible neuroprotective agent. Here, it was found that the neuroprotective effects of valproic acid were dependent upon the presence of microglia and that it in fact promoted expression of P2X7, the receptor that mediates TNF-α release upon excitotoxic insult [71]. 

## 5. Conclusions

It is an exciting time for research involving microglia, as knowledge on the phenotypes and functions of this glial cell type in both healthy and disease states is rapidly expanding. Here, we have discussed what is currently known about the roles microglia can play in MS and RE, two CNS autoimmune conditions that are also neurodegenerative states. Though microglia can contribute to further neuronal destruction, they can also be a major player in repair and prevention of damage. Further research into the neuroprotective capabilities of microglia and how pharmacological agents can be used to induce these protective phenotypes has the potential to hugely impact treatments for not only MS and RE, but other neurodegenerative and inflammatory CNS conditions as well. 

## Figures and Tables

**Table 1:** Summary of translocator protein-positron emission tomography (TSPO-PET) studies in CNS autoimmune disorders. RE: Rasmussen’s encephalitis; MS: Multiple Sclerosis.

Source	CNS Autoimmune Disorder	Radioligand	Notable Findings
Banati, et al., 1999 [26]	RE	^11^C-PK11195	Widespread radioligand uptake throughout entire affected hemisphere in patient with higher clinically severe disease.
Banati, et al., 2000 [25]	MS	^11^C-PK11195	Maximal radioligand binding in plaque areas; increased binding also detected in normal-appearing white matter (NAWM) and gray matter in post-mortem MS tissue.
Versijpt, et al., 2005 [27]	MS	^11^C-PK11195	Radioligand uptake in NAWM correlated with brain atrophy; brain atrophy correlated with disease duration and severity.
Ratchford, et al., 2012 [28]	MS	^11^C-PK11195	Radioligand binding was decreased in white matter and gray matter after one year of treatment with glatiramer acetate.
Herranz, et al., 2016 [29]	MS	^11^C-PBR28	Increased radioligand binding in the cortex, deep gray matter, and NAWM correlated with neurological disability and impaired cognition; radioligand uptake was greater in secondary progressive multiple sclerosis (SPMS) patients compared to relapsing-remitting multiple sclerosis (RRMS) patients.
